# Determining the optimum mixture of three essential oils for potato sprout suppression at room temperature storage

**DOI:** 10.3389/fpls.2023.1199117

**Published:** 2023-06-14

**Authors:** Jena L. Thoma, Charles L. Cantrell, Prabin Tamang, Valtcho D. Zheljazkov

**Affiliations:** ^1^ Department of Crop and Soil Science, Oregon State University, Corvallis, OR, United States; ^2^ Natural Products Utilization Research Unit, Agricultural Research Service, United States Department of Agriculture, University, MS, United States

**Keywords:** potato storage, essential oils, sprout suppressant, organic agriculture, room temperature, fungicide

## Abstract

As a staple crop, potatoes (*Solanum tuberosum*) play an important role in meeting daily caloric needs. To ensure adequate supplies for year-round consumption, potato quality must be maintained throughout lengthy storage periods. Towards this end, potato sprouting during storage must be minimized. Due to changing regulations regarding chemical means of potato sprout suppression, increased focus has turned to alternative products including essential oils (EO) as sprout suppressants in recent years. The complex composition of various EOs promises numerous options for sprout suppression. Furthermore, blends of several EOs may achieve enhanced sprout suppressant properties if synergistic interactions are present. We evaluated *Syzygium aromaticum*, *Artemisia herba-alba*, and *Laurus nobilis* EOs and blends thereof as sprout suppressants in potato cultivar Ranger Russet stored at room temperature and also tested for their antifungal activity against *Colletotrichum fragariae*, a causal organism of anthracnose disease in strawberries including other vegetables and fruits. *A. herba-alba* EO was an effective sprout suppressant when used alone and suppressed sprouting over the 90-day storage period. Interactions between *A. herba-alba* and *S. aromaticum* affected sprout length whereas interactions between *A. herba-alba* and *L. nobilis* EOs affected sprout number. An optimum blend of 50% - 82.31% *A. herba-alba*, 17.69% - 50% *L. nobilis*, and 0% - 1.01% *S. aromaticum* EOs could more effectively minimize tuber sprout length and number than any of the three whole EOs used alone. Among these three EOs, only *S. aromaticum* EO showed antifungal activity against *C. fragariae* in bioautography assay. These results exhibit the potential of EOs blends as a novel tactic in potato sprout suppression as well as potential natural product-based fungicides in managing *C. fragariae*.

## 1 Introduction

Cultivated in over 100 countries, potato (*Solanum tuberosum*) is the most important non-cereal crop globally (Boivin et al., 2020). A staple in the diets of many, potatoes can be sold fresh or processed into numerous products (Plant Production and Protection Division, 2009). 2019 saw the production of over 370 million tons of potato tubers, 19 million of which were produced in the United States (FAO, 2021). Sales of U.S. grown potatoes exceeded $3.6 billion in 2020, demonstrating the crop’s national and global significance (USDA NASS, 2021).

It is common for potatoes to be stored for several months before use (Paul et al., 2016a). Immediately after harvest, potatoes are in a natural state of dormancy and will not sprout for several days to weeks, depending on environmental, physiological, and hormonal factors as well as cultivar (Sonnewald & Sonnewald, 2014; Visse-Mansiaux et al., 2022). Unfortunately, the length of this innate dormancy is often unable to meet market needs (Boivin et al., 2020). Extended storage times are associated with increased water loss, disease occurrence, and sprouting in stored tubers (Magdalena & Dariusz, 2018). Moreover, appropriate management of sprouting is necessary as sprouted tubers show undesirable changes in weight, texture, and nutritional value, and sprouting is associated with formation of toxic alkaloids such as solanine (Sorce et al., 2005; Sonnewald & Sonnewald, 2014). Because sprouted potatoes are considered undesirable and inedible, sprouting during storage leads to lost revenue and increased food waste (Plant Production and Protection Division, 2009).

Strategies including storage at low temperatures and the use of chemical sprout suppressants are regularly implemented to limit tuber sprouting. For example, processing potato quality may be preserved for up to 9 months when stored between 8-12°C at 85–90% relative humidity (Paul et al., 2016a). However, these conditions alone are unable to completely suppress sprouting and sprout elongation once the period of innate dormancy has ended (Paul et al., 2016a). Though storage between 0-7°C is possible, these temperatures are known to alter reducing sugar content within the potato flesh. This is associated with darkening of potato products and the formation of acrylamide during frying (Wiltshire & Cobb, 1996). While this darkening is considered undesirable, the potential toxicity of acrylamide to humans makes cold storage of processing potatoes inadvisable (Paul et al., 2016b). Therefore, chemical sprout suppressants are commonly combined with low temperature storage to achieve adequate sprout control.

Isopropyl N-(3-chlorophenyl) carbamate (chlorpropham or CIPC) is the most widely used chemical sprout suppressant since the mid-20th century (Boivin et al., 2020). Its relatively low cost and high efficacy underlies its popularity; a single application can achieve complete sprout suppression for up to 5 months (Vaughn & Lehnen, 1991; Kleinkopf et al., 2003). However, due to environmental and health concerns of CIPC and its metabolites, CIPC was banned in the European Union in 2020 (Authority (EFSA) et al., 2017; Briddon & Stroud, 2019). While CIPC is still a legal sprout suppressant in many countries including the United States, the domestic potato industry could suffer considerable economic losses if American-grown potatoes are unable to be sold to countries with zero-tolerance policies for CIPC residues. Meanwhile, the growing popularity of organically produced foods and products in recent years suggests significant emerging economic opportunities in the potato industry; organic sales totaled $62 billion in the U.S. in 2021 (Organic Trade Association, 2022). Several essential oil (EO) sprout suppressants are commercially available including Biox-M, Biox-C, and Talent^®^. Biox-M contains 100% spearmint (*Mentha spicata* L.) EO, whereas Biox-C and Talent^®^ contain 100% clove (*Syzygium aromaticum* L.) and caraway (*Carum carvi* L.) EOs, respectively (Boivin et al., 2020). Because EOs can be produced organically, the potential for their expanded use as alternative chemical sprout suppressants in potato storage is high. Greater use of these alternatives could reduce the economic strain that recent regulations may have on the U.S. potato industry while also providing organic potato growers and processors more control over sprouting in their operations.

As plant EOs are often composed of numerous constituents, it is possible that multiple compounds may act synergistically to achieve sprout suppression. It follows that blends of different EOs may achieve better sprout suppression than a single EO used alone. This study was conducted to determine the optimum blend of clove (*Syzygium aromaticum*), armoise (*Artemisia herba-alba*), and bay laurel (*Laurus nobilis*) EOs to maximize sprout suppression in one cultivar, Ranger Russet, stored at room temperature. These EOs were chosen based on preliminary experiments showing sprout suppressant capabilities of *A. herba-alba* and *L. nobilis* EOs and the commercial use of *S. aromaticum* EO in sprout suppressant formulations.

## 2 Materials and methods

### 2.1 Plant material

Potato tubers of cultivar Ranger Russet were obtained from Hermiston Agricultural Research and Extension Center in Hermiston, OR, USA. Potatoes were harvested in September 2021 and subsequently stored in 22.5 kg mesh bags at 4°C before use in January 2022. All tubers were left untreated by any chemicals prior to the experiment.

### 2.2 Whole essential oils and EO blends

Essential oils (EOs) of *Syzygium aromaticum*, *Artemisia herba-alba*, and *Laurus nobilis* were used. *A. herba-alba* and *L. nobilis* EOs were purchased from Mountain Rose Herbs, Eugene, Oregon, USA, https://mountainroseherbs.com/. *S. aromaticum* EO was purchased from The Essential Oil Company, Milwaukie, Oregon, USA, https://www.essentialoil.com/collections/essential-oils/. Essential oil blends were created according to the design in **Table 1**.

**Table 1:** Mixture design with 3 components (*S. aromaticum* EO: EO34, *A. herba-alba* EO: EO167, *L. nobilis* EO: EO169).

Formulation code	EO34	EO167	EO169
**1**	100	0	0
**2**	0	100	0
**3**	0	0	100
**4**	50	50	0
**5**	50	0	50
**6**	0	50	50
**7**	33.33	33.33	33.33
**8**	66.67	16.67	16.67
**9**	16.67	66.67	16.67
**10**	16.67	16.67	66.67
**11**	33.33	33.33	33.33
**12**	33.33	33.33	33.33
**13**	33.33	33.33	33.33
**14**	33.33	33.33	33.33
**15**	33.33	33.33	33.33

### 2.3 Tuber treatment and storage conditions

Tubers were treated with essential oils (EOs) and EO blends and stored in a similar manner as previously described by Thoma et al. (2022). One (1) mL of each whole EO or EO blend was placed on to a cotton ball sitting in a plastic Petri dish lined with filter paper in the center of a new black 20 L plastic container. A control with 1 mL distilled water was included. Four randomly selected tubers were placed in each container after which the containers were sealed with aluminum foil for fumigation with the EO vapor. The Petri dish holding the EO had no direct contact with the tubers. Containers were topped with lids, stacked, and left undisturbed at room temperature. The experiment lasted 90 days.

#### 2.3.1 Observations

Sprout length and number of sprouts were used to evaluate treatment effects. Sprout length was measured by recording the longest sprout on each tuber in millimeters. Sprout number was measured as the total number of germinated (≥ 1 mm) eyes on each tuber. Observations were taken at 30, 45, 60, 75, and 90 days of storage. Averages for both observations were calculated for each replication and used in mixture design analysis.

### 2.4 Mixture design and statistical analysis

R software, Version 3.6.3, was used for statistical analysis. Linear mixed models were used to analyze both sprout length and number (R Core Team, 2021). To address wide variability in the data and to satisfy the assumptions for ANOVA, a square root transformation was applied to the sprout number data to achieve normality of residuals. Multiple comparisons were made using a *post-hoc* Tukey’s HSD test to identify differences in sprout length and number by treatment across all time points. Estimated marginal means and confidence intervals for the sprout number data were back-transformed for reporting and graphics. Multiple R-packages (“tidyverse”, “ggpubr”, “rstatix”, “nlme”, “emmeans”, and “ggplot2”) were used in the analysis, data summary, and graphics (Kassambara, 2020, 2020, p. 2, Kassambara, 2021; Wickham and RStudio, 2021; Lenth et al., 2022; Pinheiro et al., 2022).

Analysis of the simplex centroid mixture design was conducted using Minitab statistical software (version 16, Minitab Inc.) The three EOs (*S. aromaticum*, *A. herba-alba*, and *L. nobilis*) were included as the independent variables. A mixture experiment with three components has a simplex structure and is depicted as a triangle. The composition of each mixture can be displayed on a triangular region with its composition indicated by its position. For example, pure, single ingredients are located at the vertices of the triangle (BahramParvar et al., 2015). Component combinations in all mixtures must have the same final weight (EO34+EO167+EO169 = 100). The experimental design is shown in **Table 1**. The studied responses were sprout length and sprout number.

### 2.5 Direct bioautography bioassay

The antifungal activity of the three EOs: *S. aromaticum*, *A. herba-alba*, and *L. nobilis* against *Colletotrichum fragariae*, a causal organisms of anthracnose disease in strawberries was evaluated with the direct bioautography assay. The inoculum was prepared as described previously. Briefly, fresh conidia of *C. fragariae* was harvested from a 7-10 days old fungal culture grown on a ½ strength potato dextrose agar (PDA) by flooding the plate with 10 mL sterile water. The spore suspension was filtered through a sterile double Mira cloth (Calbiochem-Novabiochem Corp., La Jolla CA) and was centrifuged at 1968 rcf for 10 min. The supernatant was discarded, and the spores were counted in Countess 3 (Invitrogen). The spore concentration was adjusted to 3 x 105 spores mL-1 with the required volume of PDB-TLC media (12.5 g PDB, 0.5 g agar, 0.5 ml tween 80 in 500 ml water) to obtain the inoculum for bioautography assay.

A 10 µL volume of each EOs in ethyl alcohol (10 mg mL^-1^) was spotted twice onto a silica gel plate (Analtech, Inc. Silica Gel GHLF, 250 micron). The inoculum was then sprayed uniformly onto the spotted silica gel plate with a hand sprayer. The plate was placed in a moisture chamber (99.9% humidity) and incubated at ~26 °C for 4 days. The antifungal activity was evaluated with the presence of clear zones (no fungal growth) on the TLC plate. One µL volume of technical grade fungicides captan (>98%, Chem Service, Inc., West Chester, PA), and fluodioxonil (>99.5%, Chem Service, Inc., West Chester, PA) (stock concentration 2 mg/mL in ethanol) were used as a positive control.

### 2.6 Gas chromatography mass spectrometry flame ionization detection essential oil analysis

Gas chromatography (GC)—mass spectroscopy (MS)—flame ionization detection (FID) analysis of *Syzygium aromaticum*, *Artemisia herba-alba*, and *Laurus nobilis* EOs was performed by dissolving 50 μL of oil (weight also recorded) from each sample in chloroform in a 10 mL volumetric flask. Oil samples were analyzed by GC–MS–FID on an Agilent (Santa Clara, CA, USA) 7890A GC system coupled to an Agilent 5975C inert XL MSD as previously described by Thoma et al., 2022. Compounds were identified by Kovats Index analyses, direct comparison of MS and retention time to authentic standards, and comparison of mass spectra with those reported in the Adams and NIST mass spectra databases, unless otherwise noted in **Tables 2**–**4**. Commercial standards were obtained from Sigma-Aldrich (St. Louis, MO, USA) and used for direct comparison with retention time and MS data providing unequivocal identification where noted in **Tables 2**–**4**.

**Table 2:** *L. nobilis* EO (#169) constituents identified *via* GC–MS–FID analysis.

No.	Compound Name	Retention Time (min)	Calculated KI	Literature KI	Identification Methods^b^	Area %^a^
1	α-Thujene	5.8	924	924	Kovat, NIST, Adams	0.3
2	α-Pinene	6.0	932	932	Kovat, NIST, Adams, commercial standard	8.7
3	Camphene	6.4	947	946	Kovat, NIST, Adams, commercial standard	1.3
4	Sabinene	7.1	970	969	Kovat, NIST, Adams, commercial standard	5.8
5	β-Pinene	7.3	974	974	Kovat, NIST, Adams, commercial standard	3.1
6	Myrcene	7.6	986	988	Kovat, NIST, Adams, commercial standard	0.8
7	α-Phellandrene	8.1	999	1002	Kovat, NIST, Adams, commercial standard	0.4
8	α-Terpinene	8.5	1012	1014	Kovat, NIST, Adams, commercial standard	0.3
9	Para cymene	8.8	1022	1020	Kovat, NIST, Adams, commercial standard	2.5
10	Eucalyptol (1,8-cineole)	9.1	1030	1026	Kovat, NIST, Adams, commercial standard	41.0
11	γ-Terpinene	10.0	1058	1054	Kovat, NIST, Adams, commercial standard	1.3
12	Linalool	11.7	1101	1095	Kovat, NIST, Adams, commercial standard	8.3
13	Borneol	14.5	1169	1165	Kovat, NIST, Adams, commercial standard	0.3
14	Terpinen-4-ol	14.8	1177	1174	Kovat, NIST, Adams, commercial standard	5.2
15	α-Terpineol	15.5	1190	1186	Kovat, NIST, Adams, commercial standard	5.3
16	α-Terpinyl acetate	22.1	1348	1346	Kovat, NIST, Adams, commercial standard	6.1
17	Eugenol	22.6	1358	1356	Kovat, NIST, Adams, commercial standard	2.1
18	β-Elemene	23.9	1387	1389	Kovat, NIST, Adams	0.6
19	Methyl eugenol	24.4	1398	1403	Kovat, NIST, Adams, commercial standard	2.6
20	(E)-caryophyllene	25.0	1414	1417	Kovat, NIST, Adams, commercial standard	3.4
21	Aromadendrene	25.8	1436	1439	Kovat, NIST, Adams	0.5

a Area percentage determined from FID data.

b NIST, National Institutes of Standards and Technology mass spectrometry (MS) database; Kovat, Kovat retention index; Adams, Adams MS library.

**Table 3:** A*. herba-alba* EO (#167) constituents identified *via* GC–MS–FID analysis.

No.	Compound Name	Retention Time (min)	Calculated KI	Literature KI	Identification Methods^b^	Area %^a^
1	Tricyclene	5.8	924	926	Kovat, NIST, Adams, Commercial Standard	0.1
2	Camphene	6.5	948	954	Kovat, NIST, Adams, Commercial Standard	1.3
3	Sabinene	7.2	972	975	Kovat, NIST, Adams, Commercial Standard	2.0
4	α-Terpinene	8.5	1013	1017	Kovat, NIST, Adams, Commercial Standard	0.1
5	p-Cymene	8.8	1022	1024	Kovat, NIST, Adams, Commercial Standard	0.5
6	Eucalyptol	9.0	1030	1031	Kovat, NIST, Adams, Commercial Standard	0.4
7	γ-Terpinene	10.0	1059	1059	Kovat, NIST, Adams, Commercial Standard	0.2
8	α-Thujone	12.0	1112	1102	Kovat, NIST, Adams, Commercial Standard	63.6
9	β-Thujone	12.4	1120	1114	Kovat, NIST, Adams, Commercial Standard	8.5
10	Unknown	12.4	–	–	–	0.8
11	Trans-pinocarveol	13.3	1142	1139	Kovat, NIST, Adams	0.4
12	(R)-Camphor	13.4	1146	1146	Kovat, NIST, Adams, Commercial Standard	6.9
13	Sabina ketone	13.9	1158	1159	Kovat, NIST, Adams	0.3
14	Endo-borneol	14.4	1168	1169	Kovat, NIST, Adams, Commercial Standard	0.3
15	Terpinen-4-ol	14.8	1177	1177	Kovat, NIST, Adams, Commercial Standard	0.4
16	Myrtenal	15.5	1192	1195	Kovat, NIST, Adams, Commercial Standard	0.1
17	Cuminaldehyde	17.4	1239	1241	Kovat, NIST, Adams, Commercial Standard	0.3
18	Piperitone	18.0	1254	1252	Kovat, NIST, Adams, Commercial Standard	0.1
19	Germacrene D	27.5	1481	1481	Kovat, NIST, Adams	0.1
20	Unknown	38.2	–	–	–	0.7
21	Unknown	41.6	–	–	–	0.7
22	Hexadecanoic acid	45.2	1973	1959	Kovat, NIST, Adams	12.2

a Area percentage determined from FID data.

b NIST, National Institutes of Standards and Technology mass spectrometry (MS) database; Kovat, Kovat retention index; Adams, Adams MS library.

**Table 4:** *S. aromaticum* EO (#PP34) constituents identified via GC–MS–FID analysis.

No.	Compound Name	Retention Time (min)	Calculated KI	Literature KI	Identification Methods^b^	Area %^a^
1	Eucalyptol	9.0	1028	1026	Kovat, NIST, Adams, commercial standard	0.2
2	Diethyl malonate	10.6	1075	1069	Kovat, NIST, Adams, commercial standard	36.4
3	Camphor	13.4	1144	1141	Kovat, NIST, Adams, commercial standard	0.1
4	(*E*)- Cinnamaldehyde	18.9	1275	1267	Kovat, NIST, Adams, commercial standard	19.2
5	Eugenol	22.7	1362	1356	Kovat, NIST, Adams, commercial standard	36.0
6	Caryophyllene	25.1	1415	1408	Kovat, NIST, Adams, commercial standard	7.0
7	*α*-Humulene	26.4	1452	1452	Kovat, NIST, Adams, commercial standard	0.9
8	Caryophyllene oxide	31.5	1577	1583	Kovat, NIST, Adams, commercial standard	0.2

a Area percentage determined from FID data.

b NIST, National Institutes of Standards and Technology mass spectrometry (MS) database; Kovat, Kovat retention index; Adams, Adams MS library.

Compounds were quantified by performing area percentage calculations based on the total combined FID area. For example, the area for each reported peak was divided by total integrated area from the FID chromatogram from all reported peaks and multiplied by 100 to arrive at a percentage. The percentage of a peak is a percentage relative to all other constituents integrated in the FID chromatogram.

## 3 Results and discussion

### 3.1 Effects of whole essential oils on tuber sprout length and number

**Figure 1:**
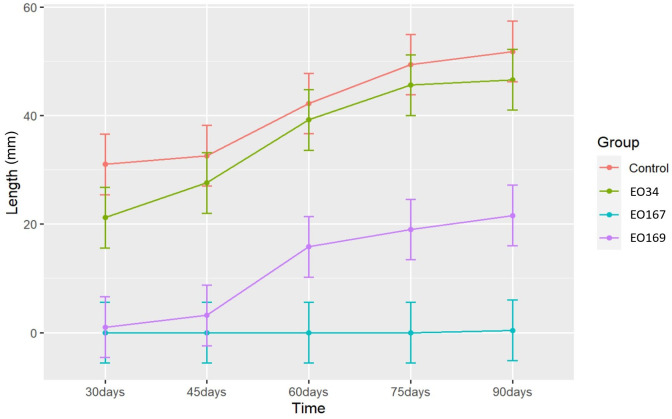
Sprout length (mm) over time of potatoes treated with distilled water (control), *S. aromaticum* (EO34), *A. herba-alba* (EO167), and *L. nobilis* (EO169) EOs. Error bars represent the 95% confidence level of the estimated means (*emmeans method*).

**Figure 2:**
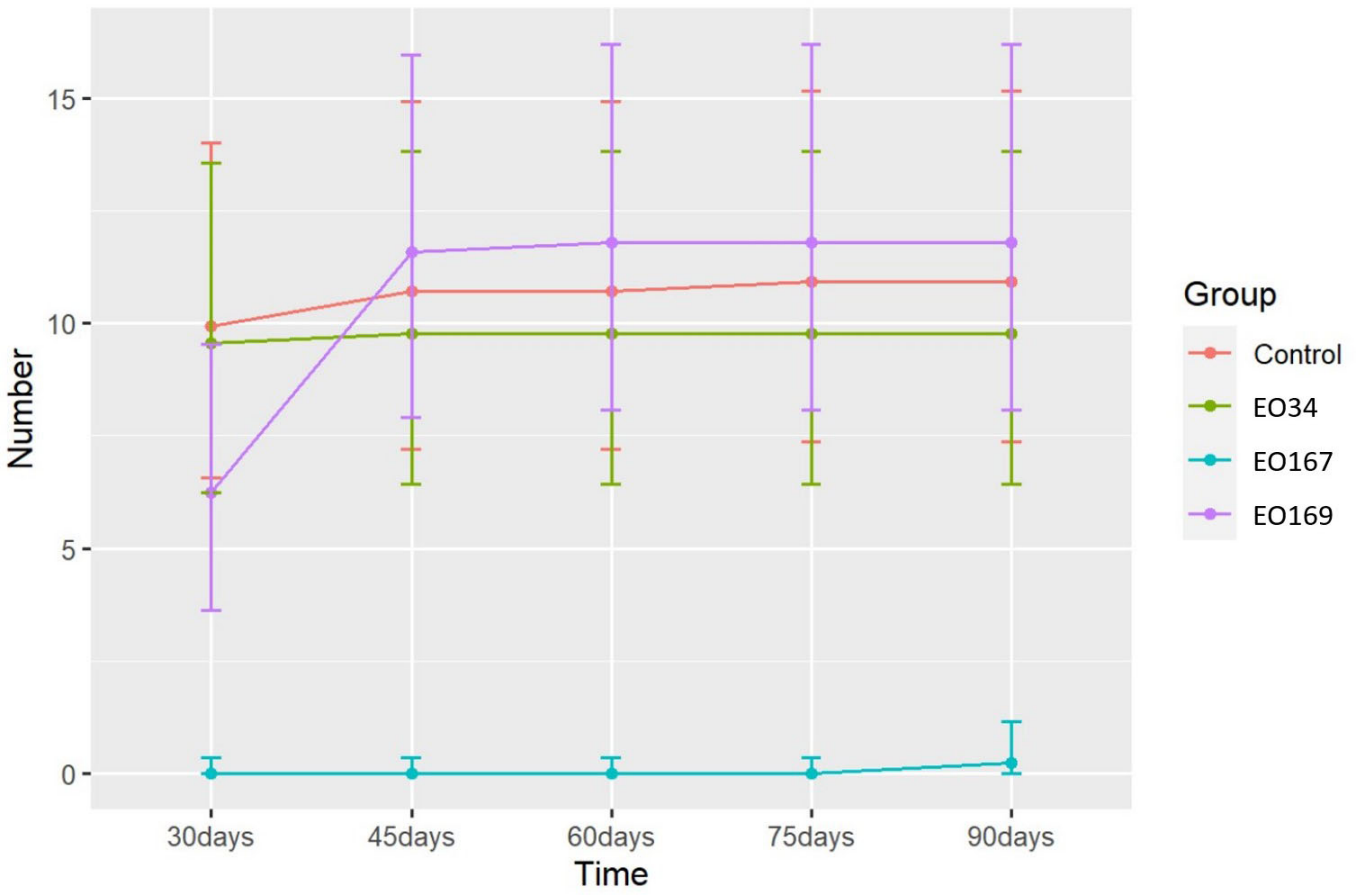
Number of germinated eyes over time of potatoes treated with distilled water (control), *S. aromaticum* (EO34), *A. herba-alba* (EO167), and *L. nobilis* (EO169) EOs. Error bars represent the 95% confidence level of the back-transformed means (*emmeans method*).

The EOs of *A. herba-alba* and *L. nobilis* significantly suppressed sprout length relative to the control throughout the 90-day storage period (**Figure 1**). *Artemisia herba-alba* EO was the most effective at reducing sprout length, limiting this response to less than 1 mm for up to 90 days (**Table 5**). *Laurus nobilis* EO limited sprout length to 3.2 mm for up to 45 days (**Table 5**). Only *A. herba-alba* EO significantly reduced sprout number, limiting this response to less than 1 germinated sprout per tuber for up to 90 days (**Table 6**). *Syzygium aromaticum* EO treatment did not significantly affect either sprout length or sprout number relative to the control at any time point (**Figures 1**, **2**).

**Table 5:** Longest sprout length (mm) of potato tubers treated with different essential oils at different time points.

Treatments	30 days	45 days	60 days	75 days	90 days
Control	31.0^a^	32.6^a^	42.2^a^	49.4^a^	51.8^a^
*S. aromaticum*	21.2^a^	27.6^a^	39.2^a^	45.6^a^	46.6^a^
*A. herba-alba*	0^b^	0^b^	0^b^	0^b^	0.4^b^
*L. nobilis*	1.0^b^	3.2^b^	15.8^c^	19.0^c^	21.6^c^

Different letters (a-c) within columns indicate statistically significant differences between treatments (Tukey’s test p < 0.05). Values are the estimated means (*emmeans method*).

**Table 6:** Number of germinated eyes of potato tubers treated with different essential oils at different time points.

Treatments	30 days	45 days	60 days	75 days	90 days
Control	9.95^a^	10.72^a^	10.72^a^	10.93^a^	10.93^a^
*S. aromaticum*	9.55^a^	9.78^a^	9.78^a^	9.78^a^	9.78^a^
*A. herba-alba*	0^b^	0^b^	0^b^	0.23^b^	0.26^b^
*L. nobilis*	6.23^a^	11.59^a^	11.8^a^	11.8^a^	11.8^a^

Values are the back-transformed means (emmeans method).

Different letters (a, b) within columns indicate statistically significant differences between treatments (Tukey’s test p < 0.05).

### 3.2 Effects of essential oil blends on tuber sprout length and number

ANOVA results and relevant p-values for the different models fitted to the responses are summarized in **Tables 7**, **8**. A significant interaction between *S. aromaticum* and *A. herba-alba* EOs on sprout length was observed up to 75 days (p ≤ 0.05), whereas a significant interaction between *A. herba-alba* and *L. nobilis* EOs on sprout length was observed at 60 days (**Table 7**). A significant interaction between all three EOs on sprout length was observed at 45 days (p ≤ 0.05) (**Table 7**). A significant interaction between *A. herba-alba* and *S. aromaticum* EOs was observed on sprout number for up to 45 days (p ≤ 0.05), whereas a significant interaction between *A. herba-alba* and *L. nobilis* EOs was observed for up to 75 days (**Table 8**).

**Table 7:** Analysis of variance for the different models fitted to the sprout length data. EO34 corresponds to *S. aromaticum* EO, EO167 corresponds to *A. herba-alba* EO, and EO169 corresponds to *L. nobilis* EO.

	30 days			45 days			60 days			75 days			90 days		
**Variation source**	df	SeqSS	P	df	SeqSS	P	df	SeqSS	P	df	SeqSS	P	df	SeqSS	P
**Regression**	5	459.994	0.001	6	921.179	0	5	1833.98	0	5	2346.56	0.001	5	2318.94	0.013
**Linear**	2	300.876	0.001	2	614.512	0	2	1202.95	0	2	1652.23	0.002	2	1729.86	0.012
**Quadratic**	3	159.118	0.007	3	282.019	0.001	3	631.02	0	3	694.33	0.018	3	589.09	0.137
EO34*EO167	1	154.199	0.001	1	262.256	0	1	468.41	0	1	540.13	0.01	1	489.34	0.055
EO34*EO169	1	0.538	0.858	1	1.004	0.24	1	65.78	0.067	1	65.18	0.308	1	51.7	0.505
EO167*EO169	1	4.381	0.44	1	18.758	0.489	1	96.83	0.015	1	89.02	0.173	1	48.05	0.464
**Special Cubic**				1	24.468	0.018									
EO34*EO167*EO169				1	24.458	0.018									
**Residual error**	9	60.539		8	22.17		9	97.98		9	365.04		9	741.04	
**Total**	14	520.533		14	943.349		14	1931.96		14	2711.62		14	3059.98	

**Table 8:** Analysis of variance for the different models fitted to the sprout number data. EO34 corresponds to *S. aromaticum* EO, EO167 corresponds to *A. herba-alba* EO, and EO169 corresponds to *L. nobilis* EO.

	30 days			45 days			60 days			75 days			90 days		
**Variation source**	df	SeqSS	P	df	SeqSS	P	df	SeqSS	P	df	SeqSS	P	df	SeqSS	P
**Regression**	5	164.805	0	5	223.16	0	5	220.41	0	5	217.406	0.001	5	213.845	0.008
**Linear**	2	117.091	0.003	2	144.03	0.003	2	146.551	0.002	2	162.178	0.004	2	166.389	0.018
**Quadratic**	3	47.714	0.01	3	79.131	0.006	3	73.859	0.006	3	55.228	0.034	3	47.456	0.139
EO34*EO167	1	24.592	0.019	1	22.159	0.08	1	19.312	0.1	1	18.554	0.131	1	20.237	0.185
EO34*EO169	1	0.007	0.672	1	5.144	0.452	1	4.61	0.479	1	1.899	0.75	1	0.446	0.987
EO167*EO169	1	23.114	0.011	1	51.467	0.003	1	49.938	0.003	1	34.775	0.017	1	26.773	0.077
**Residual error**	9	20.624		9	28.269		9	27.179		9	36.53		9	60.337	
**Total**	14	184.429		14	251.429		14	247.589		14	253.936		14	274.182	

**Figure 3:**
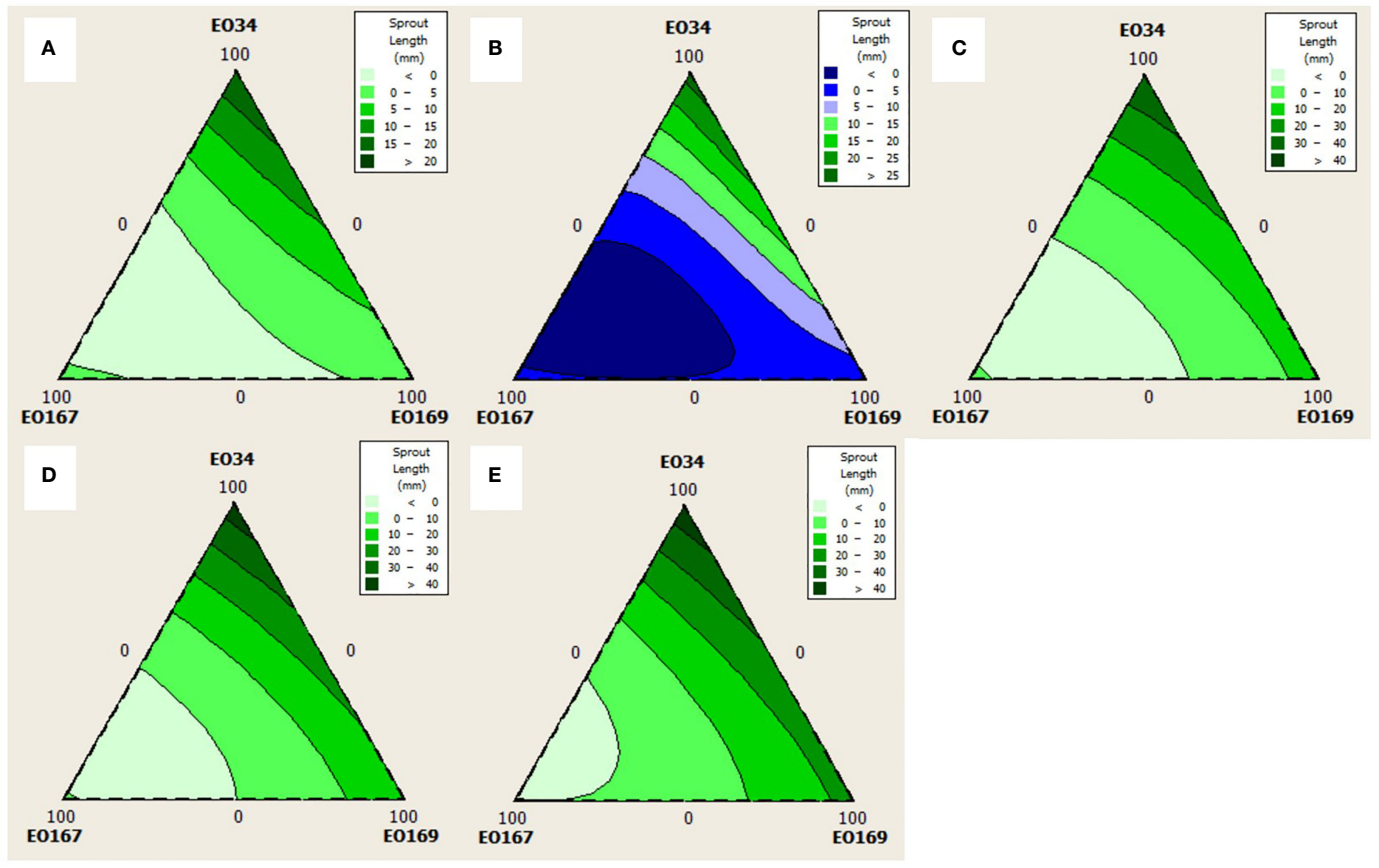
Contour plots for the effect of different combinations of *S. aromaticum* (EO34), *A herba-alba* (EO167), and *L. nobilis* (EO169) EOs on sprout length at **(A)** 30 days, **(B)** 45 days, **(C)** 60 days, **(D)** 75 days, and **(E)** 90 days.

**Figure 4:**
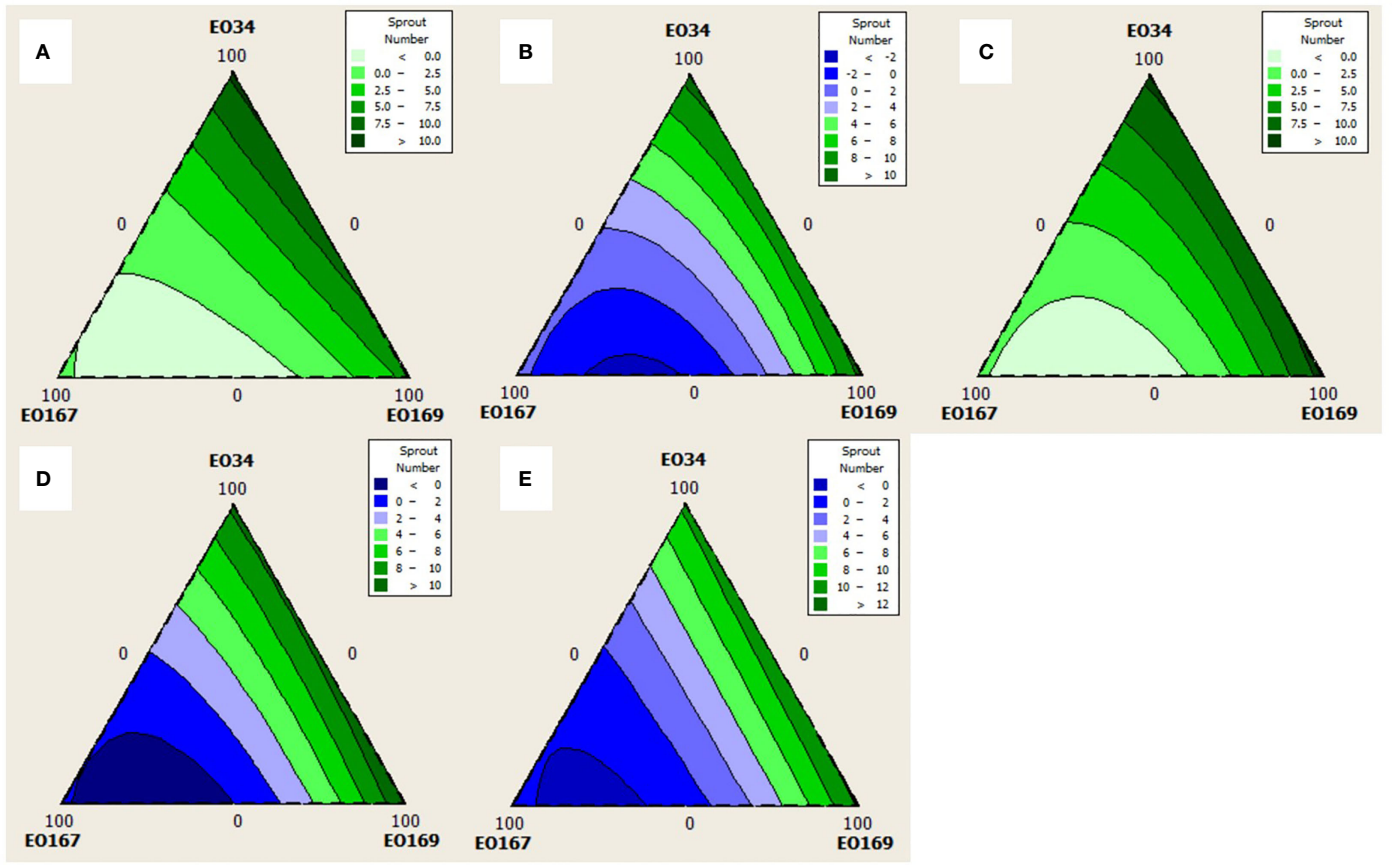
Contour plots for the effect of different combinations of *S. aromaticum* (EO34), *A herba-alba* (EO167), and *L. nobilis* (EO169) EOs on sprout number at **(A)** 30 days, **(B)** 45 days, **(C)** 60 days, **(D)** 75 days, and **(E)** 90 days.

Quadratic models were chosen for analysis of sprout number at all time points as simple cubic models were not significant (not shown, p > 0.05). Quadratic models were chosen for analysis of sprout length for every time point except 45 days for which a simple cubic model was used. The triangular mixture contour plots of the responses are depicted in **Figures 3**, **4** to display the interaction between components on sprout length and number, respectively, across time.

### 3.3 Formulation optimization

A response optimizer was used to identify the optimum blend to minimize both responses at the various time points. The parameters used in the optimization are shown in **Table 9**. Minimization of both sprout length and number was preferred. The optimum blend consists of 50% - 82.31% *A. herba-alba* EO, 0% - 1.01% *S. aromaticum* EO, and 17.69% - 50% *L. nobilis* EO depending on storage length (**Table 10**).

**Table 9:** The desirable ranges for each response to determine the optimum EO blend at each time point.

	Parameters	Goal	Lower	Target	Upper	Weight	Importance
**30 days**	Sprout length	Minimum	0	0	21.2	1	1
	Sprout number	Minimum	0	0	10.6	1	1
**45 days**	Sprout length	Minimum	0	0	27.6	1	1
	Sprout number	Minimum	0	0	11.6	1	1
**60 days**	Sprout length	Minimum	0	0	39.2	1	1
	Sprout number	Minimum	0	0	11.8	1	1
**75 days**	Sprout length	Minimum	0	0	45.6	1	1
	Sprout number	Minimum	0	0	11.8	1	1
**90 days**	Sprout length	Minimum	0.4	0.4	46.6	1	1
	Sprout number	Minimum	0.2	0.2	11.8	1	1

**Table 10:** Proportions of each EO predicted to achieve optimal suppression of both sprout length and number at each time point.

	*S. aromaticum*	*A. herba-alba*	*L. nobilis*
**30 days**	0	50	50
**45 days**	1.01	66.64	32.35
**60 days**	0	65.66	34.34
**75 days**	0	72.73	27.27
**90 days**	0	82.31	17.69

### 3.4 Gas chromatography– mass spectroscopy– flame ionization detection

Results of gas chromatography (GC) – mass spectroscopy (MS) – flame ionization detection (FID) analysis of *A. herba-alba*, *S. aromaticum*, and *L. nobilis* EOs are presented in **Tables 2**–**4**, respectively. Compounds making up > 1% of *A. herba-alba* EO included α-thujone (63.6%), β-thujone (8.5%), hexadecenoic acid (12.2%), (R)-camphor (6.9%), sabinene (2.0%), and camphene (1.3%) (**Table 3**). Compounds making up > 1% of *S. aromaticum* EO included diethyl malonate (36.4%), eugenol (36%), (E)-cinnamaldehyde (19.2%), and caryophyllene (7.0%) (**Table 4**). Compounds making up > 1% of *L. nobilis* EO included eucalyptol (41.0%), α-pinene (8.7%), linalool (8.3%), α-terpinyl acetate (6.1%), sabinene (5.8%), α-terpineol (5.3%), terpinene-4-ol (5.2%), (E)- caryophyllene (3.4%), β-pinene (3.1%), methyl eugenol (2.6%), para cymene (2.5%), eugenol (2.1%), camphene (1.3%), and γ-terpinene (1.3%) (**Table 2**).

### 3.5 Bioautography assay

**Figure 5:**
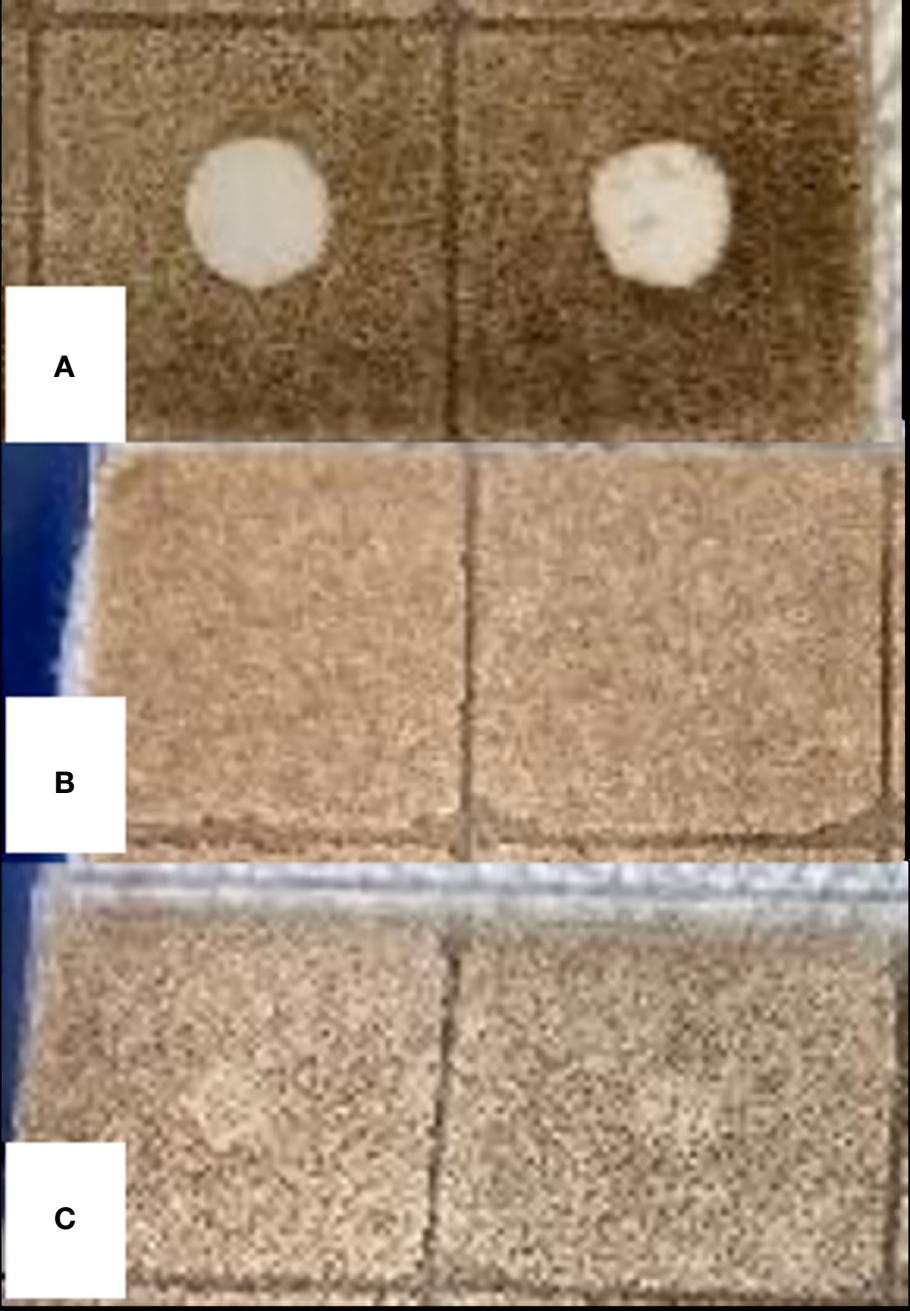
Bioautography assay of EOs from *S. aromaticum* **(A)**, *A herba-alba* **(B)**, and *L. nobilis* **(C)**. The antifungal activity against *C fragariae* is represented by “zone of fungal growth inhibition”.

Among the three whole EOs, only *S. aromaticum* EO showed antifungal activity against *C. fragariae* in bioautography assay with a clear zone of fungal growth inhibition (**Figure 5**). The average ± standard deviation from two replications was 8.3 ± 0.35 mm for *S. aromaticum* EO. The EOs from *A. herba-alba* and *L. nobilis* did not show any antifungal activity against *C. fragariae* in our study.

## 4 Discussion

Sprout length is one of many important traits used to grade potatoes in the United States. For example, processing potatoes with sprout lengths longer than half an inch (~13 mm) or one inch (~25 mm) are considered “damaged” and “seriously damaged”, respectively (USDA AMS, 2011). Potatoes with sprouts are considered defects, and greater degrees of damage result in lower grades and increased food waste (Pedreschi et al., 2008).

Sprout number is another important attribute that affects potato grading in the United States. Unlike sprout length, there is no specific limit on the allowable number of sprouts per tuber. However, if numerous individual or clusters of sprouts are present, lower potato grades may result (USDA AMS, 2011). Individual tubers possess numerous eyes from which sprouts can develop. Therefore, limiting sprout number in addition to sprout length is crucial for maintaining tuber quality.

Average longest sprout length varied between 0 and 46.6 mm depending on the EO blend with higher concentrations of *A. herba-alba* EO showing the strongest negative relationship with sprout length. A significant 3-way interaction on sprout length at 45 days suggests that a blend of the three EOs could achieve better suppression of sprout length than when any of the EOs is used alone. Indeed, response optimization suggested a blend of all three EOs for storage lengths of 45 days, though the efficacy of this blend has yet to be tested.

Average sprout number varied between 0 and 11.8, with higher concentrations of *A. herba-alba* EO showing the strongest negative relationship with sprout number. The interactions between *A. herba-alba* and *S. aromaticum* EOs and *A. herba-alba* and *L. nobilis* EOs for up to 45 days and 75 days, respectively, suggests that a combination of these EOs could obtain better control of sprout number than when any of the EOs are used alone. At all timepoints, response optimization suggests that a blend of *A. herba-alba* and *L. nobilis* EOs could result in better sprout suppression than *A. herba-alba* EO alone, although for longer storage periods, greater proportions of *A. herba-alba* EO are suggested.

Essential oils are known to contain numerous compounds, some of which have demonstrated sprout suppressant activity (Boivin et al., 2020). Of the three EOs, *L. nobilis* contained the most compounds previously reported to be effective sprout suppressants at amounts >1% including α-pinene (8.7%), eucalyptol (41.0%), and terpinen-4-ol (5.2%) (Boivin et al., 2020). *A. herba-alba* EO contained camphor (6.9%), previously reported as a somewhat effective sprout suppressant, but its primary constituent α-thujone (63.6%) has been reported as an ineffective sprout suppressant when used alone (Boivin et al., 2020). The higher efficacy of *A. herba-alba* EO over *L. nobilis* EO as a sprout suppressant could be due to synergistic interactions between its various constituents. Furthermore, the superior performance of an EO blend over any of the three EOs used individually could be due to compounds within the EOs acting synergistically.

Of the three whole EOs, *S. aromaticum* EO was the least effective as a sprout suppressant. These findings are corroborated by previous findings demonstrating *A. herba-alba* as an effective sprout suppressant in Ranger Russet potato storage (Thoma et al., 2022). Though *S. aromaticum* EO is the main component of Biox-C, a commercial sprout suppressant, treatment with *S. aromaticum* EO did not result in adequate sprout suppression in the present study. This could be due to only a single application of EO when repeated applications are often necessary, or the amount applied may have been too low.

*S. aromaticum* EO was reported to have antimicrobial properties against both fungi and bacteria (Burt and Reinders, 2003; Fu et al., 2007; Pinto et al., 2009; Gupta et al., 2014; Aguilar-González et al., 2015; Harčárová et al., 2021). Harčárová et al. (2021) has reported 100% growth inhibition in both *in-vivo* and *in-vitro* study of *Fusarium graminearum* at 500 and 1000 µg/ml. Similarly, *S. aromaticum* EO demonstrated antifungal activity with a minimum inhibitory concentration (MIC) of 92.56 µL/L_air_ against gray mold disease of fruits and vegetables caused by *Botrytis cinerea* (Aguilar-González et al., 2015). The MIC of *S. aromaticum* EO was even lower with 46.28 µL/L_air_ in *in-vitro* and 11.57 µL/L_air_ in *in-vivo* when combined with Mustard EO. Neither of these studies have further investigated the EO to determine the major bioactive constituents in *S. aromaticum*. The antifungal activity was also reported against human pathogens like *Candida* sp., *Aspergillus* sp. and dermatophyte species (Pinto et al., 2009) which reported eugenol is the major component of this EO (85.3%).

The EO from *A. herba-alba* did not show any antifungal activity against *C. fragariae* in our study. However, *A. dracunculus* L. var. *dracunculus* and *A. douglasiana* has shown antifungal activity against *C. acutatum*, *C. fragariae*, *C. gloesporioides* and *Botrytis cinerea* (Meepagala et al., 2002 and Meepagala et al., 2003). Vulgarone B was isolated from *A. douglasiana* as an active compound (Meepagala et al., 2003).

*L. nobilis* EO has been reported to have antifungal properties against post-harvest pathogens in cherry tomatoes (Xu et al., 2014), seed borne fungi of cucurbits (Moumni et al., 2021) and citrus black rot disease (Soylu and Kose, 2015) which are all caused by *Alternaria alternata*. However, its antifungal activity against *C. fragariae* has not been reported.

Additional studies verifying the ability of the optimum blend to suppress sprouting in Ranger Russet and other potato cultivars are warranted. Prior to industry adoption of this novel treatment, the optimum blend’s efficacy should be evaluated at semi-commercial or commercial scale using industrial infrastructure and application methods. In addition, pure compounds from *L. nobilis* and *A. herba-alba* EOs may be blended and tested to reveal which components are acting synergistically. Results from the present study suggest that EO blends may provide even greater sprout control than single EOs used alone. Furthermore, EO blends may even outperform commercially available EO sprout suppressant products, requiring lower concentrations or less frequent applications, which could offset the costs of using EOs in this industry. Therefore, experiments comparing this blend to currently available EO sprout suppressants and conventional products are justified.

## 5 Conclusion

As international restrictions on dominant sprout suppressant chemicals tighten, the need for effective alternatives is only expected to grow. Essential oils (EOs) have a history of use as potato sprout suppressants and may achieve varying rates of suppression depending on potato cultivar, storage temperature, and method and timing of application. Due to variability in EO compositions, it is possible that these constituents may interact to produce enhanced sprout suppression. This study demonstrated that *A. herba-alba* and *L. nobilis* EOs can significantly suppress sprouting in Ranger Russet tubers when used alone. A simplex centroid mixture design was used to determine the optimum blend of three EOs (*S. aromaticum*, *A. herba-alba*, and *L. nobilis*) to achieve sprout suppression in Ranger Russet potato tuber storage. The optimum blend to minimize both sprout length and number over a 90-day storage period consists of 50% - 82.31% *A. herba-alba* EO, 17.69% - 50% *L. nobilis* EO, and 0% - 1.01% *S. aromaticum* EO. This suggests that a blend of two or more EOs could achieve better sprout suppression than a single EO alone. Though the efficacy of this blend remains to be verified, the results illustrate the promise of mixture experiments in the development of novel sprout suppressant treatments. EO blends could provide increased control over potato sprouting and may even perform better than currently available EO sprout suppressant products. EOs therefore have a significant role to play in increasing the economic resilience of the potato industry while reducing food waste. Also, the result from bioautography assay of *S. aromaticum* EO’s exhibited antifungal activity against *C. fragariae*, and it could serve as a potential natural-product based fungicide as well as a sprout suppressant in potato.

## Data availability statement

The raw data supporting the conclusions of this article will be made available by the authors, without undue reservation.

## Author contributions

JT: Formal analysis, Investigation, Writing – original draft, Writing – review and editing, Visualization. CC: Formal analysis, Investigation, Writing – review and editing. PT: Formal analysis, Investigation, Writing – original draft, Writing – review and editing. VZ: Conceptualization, Methodology, Resources, Writing – review and editing, Supervision, Project administration, Funding acquisition. All authors contributed to the article and approved the submitted version.
